# Investigation of the Immunomodulatory and Neuroprotective Properties of *Nigella sativa* Oil in Experimental Systemic and Neuroinflammation

**DOI:** 10.3390/ijms26052235

**Published:** 2025-03-02

**Authors:** Anita Mihaylova, Nina Doncheva, Maria Vlasheva, Mariana Katsarova, Petya Gardjeva, Stela Dimitrova, Ilia Kostadinov

**Affiliations:** 1 Department of Pharmacology, Toxicology and Pharmacotherapy, Faculty of Pharmacy, Medical University of Plovdiv, 15A Vasil Aprilov Blvd., 4002 Plovdiv, Bulgaria; nina.doncheva@mu-plovdiv.bg; 2 Research Institute, Medical University of Plovdiv, 15A Vasil Aprilov Blvd., 4002 Plovdiv, Bulgaria; stela.dimitrova@mu-plovdiv.bg; 3 Department of Bioorganic Chemistry, Faculty of Pharmacy, Medical University of Plovdiv, 15A Vasil Aprilov Blvd., 4002 Plovdiv, Bulgaria; mariya.vlasheva@mu-plovdiv.bg (M.V.); mariana.katsarova@mu-plovdiv.bg (M.K.); 4 Department of Medical Microbiology and Immunology “Prof. Dr. Elissay Yanev”, Faculty of Medicine, Medical University of Plovdiv, 15A Vasil Aprilov Blvd., 4002 Plovdiv, Bulgaria; petya.gardjeva@mu-plovdiv.bg; 5 Department of Pharmacology and Clinical Pharmacology, Faculty of Medicine, Medical University of Plovdiv, 15A Vasil Aprilov Blvd., 4002 Plovdiv, Bulgaria

**Keywords:** *Nigella sativa* oil, neuroinflammation, thymoquinone, inflammatory cytokines, BDNF, memory, NPY

## Abstract

*Nigella sativa* (NS) is a promising medicinal plant with diverse therapeutic properties. This study aimed to investigate the impact of NS oil (NSO) on memory functions in rats with LPS (lipopolysaccharide)-induced neuroinflammation, as well as its effect on serum levels of inflammatory cytokines, neuropeptide Y (NPY) and brain-derived neurotrophic factor (BDNF). Male rats were divided into four groups: control, LPS-control, LPS+NSO 3 and 5 mL/kg. Neuroinflammation was induced by a single intraperitoneal LPS injection (2 mg/kg). The novel object recognition test (NORT) and Y-maze were used for the evaluation of memory processes. Recognition index (RI) and % spontaneous alteration (%SA) were registered, respectively. Blood samples for TNF-α, IL-1β, IL-10, BDNF, and NPY serum levels were taken. Thymoquinone, the active compound of the oil, was detected by high-performance liquid chromatography. NSO administration resulted in an improvement in spatial and episodic memory, as evidenced by increased % SA and RI compared to LPS-control. Treatment with NSO led to a significant reduction in pro-inflammatory cytokines and NPY, along with an increase in IL-10 and BDNF levels, when compared to LPS-control. In conclusion, NSO enhances BDNF production and regulates pro- and anti-inflammatory cytokines release, which probably contributes to the observed cognitive improvement in animals with experimental neuroinflammation.

## 1. Introduction

Inflammation is recognized as a complex physiological response initiated by diverse stimuli, including foreign particles, pathogenic microorganisms, physical injury, dysregulation of immune function, exposure to chemical agents, etc. [1]. It plays a critical role in the progression and exacerbation of numerous chronic conditions, including neurodegenerative disorders [2]. Persistent or dysregulated inflammatory responses can contribute to tissue damage, impair cellular function, and accelerate disease progression, making it a central target in understanding and managing these chronic conditions. Research indicates that systemic inflammation can induce neuroinflammation, which may initiate or worsen neurological conditions such as Alzheimer’s disease (AD), Parkinson’s disease (PD), depression, anxiety, and cognitive impairment. This inflammatory crosstalk between the immune system and the central nervous system (CNS) can disrupt neural homeostasis, leading to neurodegenerative changes and altered neurotransmitter function that underpin these disorders [3].

The immune system plays a crucial role in modulating brain function, including processes related to learning and memory. Cytokines are key mediators of both systemic and neuroinflammation. In healthy individuals, a balance between pro-inflammatory cytokines (PICs) (e.g., TNF-α, IL-1β, IL-6) and anti-inflammatory cytokines (e.g., IL-10) is maintained, supporting immune regulation and normal cognitive functions [4]. This equilibrium is essential for brain health, as pro-inflammatory cytokines can impair cognitive function if unregulated, while anti-inflammatory cytokines help limit excessive inflammatory responses that could otherwise disrupt neural circuits. Key pro-inflammatory cytokines, such as TNF-α and IL-1β, are among the first to be secreted at inflammation sites. They contribute to the detrimental effects of neuroinflammation on cognitive processes [5]. These cytokines can disrupt synaptic plasticity and neural connectivity, ultimately impairing cognitive function and contributing to the pathogenesis of neurodegenerative and cognitive disorders. TNF-α is a central player in initiating and regulating inflammation. Produced primarily by immune cells like macrophages and microglia (in the central nervous system), TNF-α plays a dual role in health and disease [6]. Excessive TNF-α has been implicated in autoimmune disorders, neurodegenerative diseases such as AD and PD, and even in mood disorders where chronic inflammation plays a role in pathogenesis [6,7,8]. In the hippocampus, TNF-α overproduction has been shown to reduce long-term potentiation (LTP) in both the CA1 region and the dentate gyrus by altering synaptic receptor composition and weakening the signaling pathways essential for LTP induction. This disrupts the synaptic modifications necessary for encoding new information, leading to impairments in learning and memory [5].

IL-1β plays a pivotal role in amplifying the inflammatory response by promoting the synthesis of secondary inflammatory molecules [1]. Microglia are the main cell population activated during neuroinflammation, resulting in an elevated release of PICs. Studies indicate that elevated serum levels of IL-1β and IL-6 are associated with significant cognitive decline, suggesting a direct link between neuroinflammatory processes and impaired cognitive function [3]. This relationship underscores the role of microglia-driven inflammation in neurodegenerative diseases and cognitive impairments, highlighting the potential impact of systemic cytokine levels on brain health. IL-10 is well recognized for its potent anti-inflammatory effects and plays a crucial role in modulating neuroinflammation by suppressing the inflammatory activity of immune cells [9]. Additionally, IL-10 promotes the synthesis of TGF-β1 by astrocytes, which further contributes to reducing neuroinflammation by dampening microglial activation [10]. Experimental studies show that IL-10 ameliorates cognitive dysfunction induced by PICs [11].

Brain-derived neurotrophic factor (BDNF) is a neurotrophin predominantly produced by glial cells, essential for neuronal survival and the maintenance of normal brain functions, including neuronal plasticity and neurotransmitter regulation [12]. It plays a critical role as a regulator of synaptic transmission and LTP, particularly within the hippocampus and other brain regions, supporting learning and memory processes. Low BDNF levels have been implicated in the pathogenesis of several neurological conditions, such as dementia, depression, schizophrenia, and anxiety disorders, where neuroinflammation is also a significant contributing factor [13]. Neuropeptide Y (NPY) is synthesized and released by both the central and peripheral nervous systems, as well as by certain immune cells [14]. Evidence suggests that NPY is involved in the pathogenesis of stress-related and neurodegenerative conditions [15,16]. It plays a regulatory role in the functioning of inflammatory cells and serves as a link between the immune and neuroendocrine systems. It stimulates immune cells and modulates the release of PICs, including TNF-α [17].

*Nigella sativa* (NS), commonly known as black cumin, is regarded as a promising medicinal plant with a wide range of therapeutic properties. It belongs to the *Ranunculacea* family and is native to Southern Europe, North Africa, and Asia, especially Syria, India, Pakistan, Turkiye, and Saudi Arabia [18,19]. It is used in traditional medicine for the treatment of various diseases and disorders such as bronchitis, diabetes, cancer, inflammation, hypertension, fever, gastrointestinal symptoms, neurological problems, etc. [20]. Existing studies indicate that NS exhibits antioxidant, anti-inflammatory, antibacterial, anxiolytic, and immunomodulatory activities [21]. *Nigella sativa* oil (NSO) is extracted from the seeds of the *Nigella sativa* plant using various methods such as cold pressing, microwave-assisted extraction, ultrasound-assisted extraction, and supercritical fluid extraction, among others. The oil contains a combination of mono- and polyunsaturated fatty acids (such as oleic and linoleic acids) and saturated fatty acids (like palmitic acid), along with sterols, carbohydrates, amino acids, flavonoids, tannins, alkaloids, terpenes, saponins, glycosides, and other compounds [22]. Phytochemical analysis of NSO has identified over 35 active compounds, including the monoterpene derivatives thymoquinone (TMQ), dithymoquinone, thymol, and carvacrol. [23]. Among these, TMQ is recognized as the primary component of the oil and is considered the most bioactive ingredient, demonstrating a variety of medicinal benefits [21]. While thymol and carvacrol are stable under different conditions, including hydrolysis, oxidation, exposure to direct sunlight, and high temperature, TMQ appears to degrade when exposed to heat and light [24]. Since TMQ is the main biologically active ingredient in NSO, developing analytical methods for its quantitative determination is important in order to predict the expected benefits of its use in the prevention and treatment of various medical conditions.

Neuroprotective and memory-improving effects of NS have been demonstrated in numerous studies. In an experimental model of pentylenetetrazole-induced seizures in rats, NS seed extracts prevent hippocampal neuron damage, improve impaired memory in the passive avoidance test [25] and the Morris water maze, and have a beneficial effect on LTP in the hippocampus [26]. Additionally, NS extract improves memory in rats with hypothyroidism-induced memory decline during the neonatal and juvenile periods, as assessed by the aforementioned paradigms [27]. Mahmoud Janloo et al. found that intraperitoneal administration of a hydro-alcoholic extract of NS seeds decreases oxidative stress in hippocampal and cortical neurons and improves spatial memory (in the Morris water maze) in Wistar rats with cisplatin-induced neurotoxicity, with the most significant effects observed at a dose of 400 mg/kg [28]. Moreover, a two-week pretreatment with hydro-alcoholic extract of NS seeds prevents memory impairment, decreases acetylcholinesterase activity, and reduces oxidative stress in rats with scopolamine-induced spatial memory impairment [29]. In LPS-induced memory impairment in rats, NS seed extract not only improves memory functions in the passive avoidance test and the Morris water maze but also decreases hippocampal levels of IL-6, TNF-α, malondialdehyde, and nitric oxide metabolites while increasing thiol content, catalase, and superoxide dismutase activity [30]. Furthermore, the extract restores LTP in the hippocampus of rats subjected to intraperitoneal LPS administration [5].

Along with the extract, NSO and TMQ are also the subject of extensive research for their neuroprotective, antioxidant, and anti-inflammatory effects. In restrained rats, NSO at a dose of 0.2 mL/kg/day improves short-term memory in the elevated plus maze, decreases acetylcholine esterase activity, and demonstrates an antioxidant effect [31]. Sahak MK et al. found that NSO improves the learning and memory of naïve rats in the radial arm maze [32]. The anti-inflammatory effects of NSO have been demonstrated in various experimental models, including autoimmune encephalomyelitis [33], carrageenan-induced paw edema, formaldehyde-induced arthritis, cotton pellet granuloma, and Freund’s adjuvant-induced paw edema [18,34]. The doses of NSO in these studies vary from 1 to 10 mL/kg. Chronic administration of TMQ (25 mg/kg) improves memory, stimulates neurogenesis, decreases oxidative stress, and increases mRNA and protein expression of BDNF in the rat hippocampus [35]. Additionally, TMQ demonstrates memory-improving and neuroprotextive effects in different models of neurotoxicity, such as nonylphenol-induced memory impairment [36], the rat model of AD [37], and D-galactose and aluminum trichloride-induced toxicity [38], among others. The neuroprotective effects of TMQ may be attributed to its antioxidant anti-inflammatory properties [39]. Since neurodegenerative disorders are strongly linked to neuroinflammation, we hypothesized that by inhibiting systemic inflammation, NSO may alleviate memory decline in a rat model of systemic, neuroinflammation, and neurodegeneration [40].

Various substances, including lipopolysaccharide (LPS), are employed to model systemic and neuroinflammation, as well as neurodegeneration. The interplay between neurodegeneration and neuronal cell death can trigger an inflammatory response, which in turn may contribute to further cell death. LPS is commonly used in experimental studies to induce inflammation and memory impairment in rodent models, allowing researchers to investigate the underlying mechanisms of neuroinflammation and its effects on cognitive function [41]. This approach helps elucidate the complex relationship between inflammation and neurodegenerative processes.

The aim of our study was to explore the potential cognitive benefits of the NSO in the context of neuroinflammation and neurodegeneration. In this regard, it was developed and validated an HPLC method for the quantification of TMQ, thymol, and carvacrol which are the main active molecules in this oil. Additionally, examining the relationship between memory improvement and the levels of inflammatory markers and neurotrophic factors could provide insights into the mechanisms behind NSO’s effects. This approach could clarify whether NSO’s potential benefits are due to its anti-inflammatory actions, its ability to support neuroplasticity or both.

## 2. Results

### 2.1. HPLC Quantification of TMQ, Thymol, and Carvacrol in NSO

An HPLC method was developed for the quantification of TMQ, thymol, and carvacrol. Parameters of calibration curves, RSD, LOD, and LOQ for HPLC method validation are shown in Table 1.

**Figure 1:**
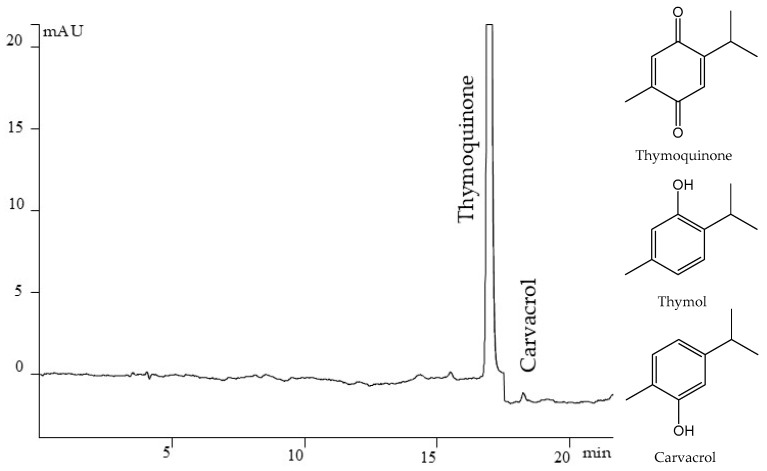
Chromatogram of NS seeds oil (1 ppm in hexane) with detected thymoquinone at 254 nm, carvacrol at 275 nm, and structures of the analyzed substances.

Determination coefficients (r^2^) between 0.9974 and 0.9996 with RSD ranging from 1.77 to 5.92% validate the linearity of this method for the quantification of thymoquinone, thymol, and carvacrol. The correlation coefficients (r) 0.9991 and 0.9989 prove the spectral similarity of the peaks of the substances of interest. The short analysis, the available equipment, and the accuracy of the proposed method enable it to be used for routine work. Through it, the quality of raw material can be controlled, as well as ready phytomedication based on Nigella sativa. The amount of the main active substance thymoquinone was 21.37 ± 0.38 mg/mL (2.137 ± 0.038%), the amount of carvacrol was in traces but thymol was not detected in the used oil. Figure 1 shows a chromatogram of NS seed oil.

### 2.2. Effects of NSO on Serum Levels of TNF-α, IL-1β, IL-10, BDNF and NPY in LPS-Challenged Rats

#### 2.2.1. TNF-α

**Figure 2:**
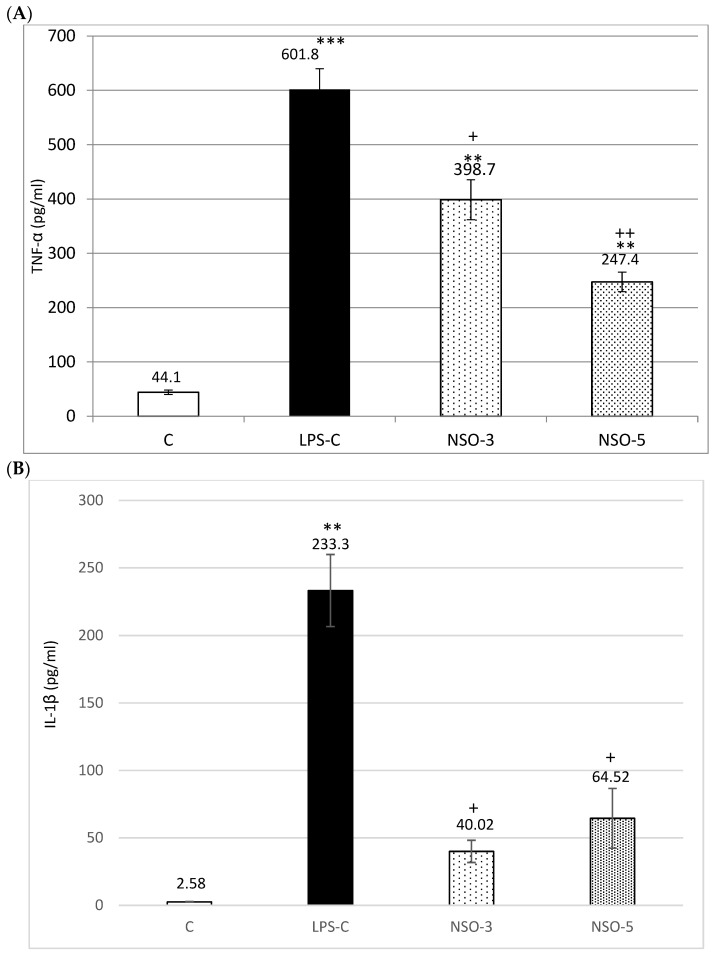

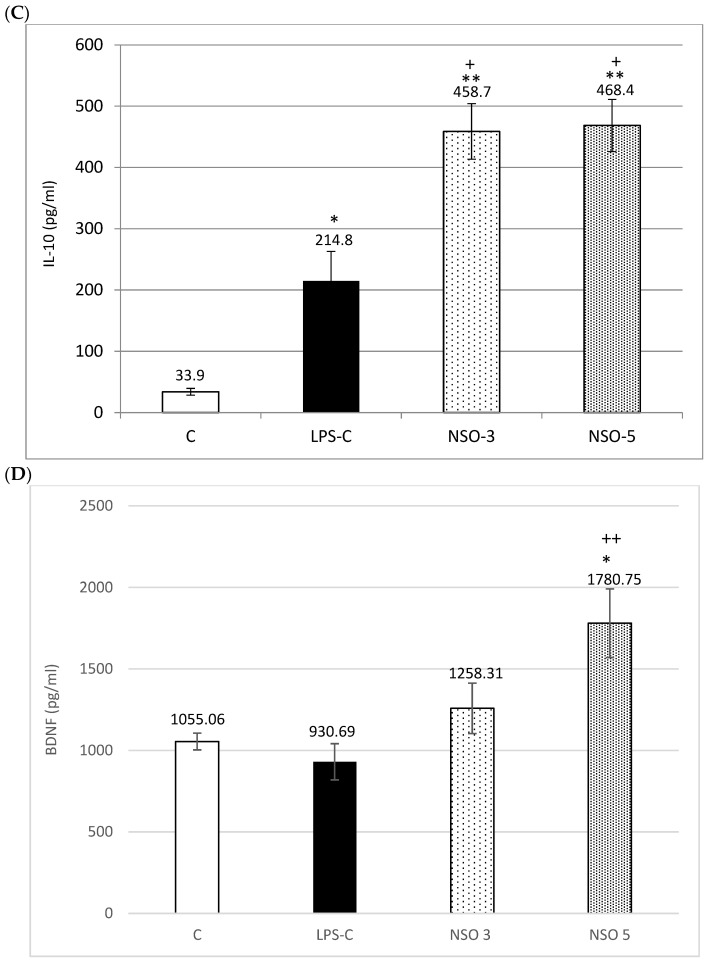

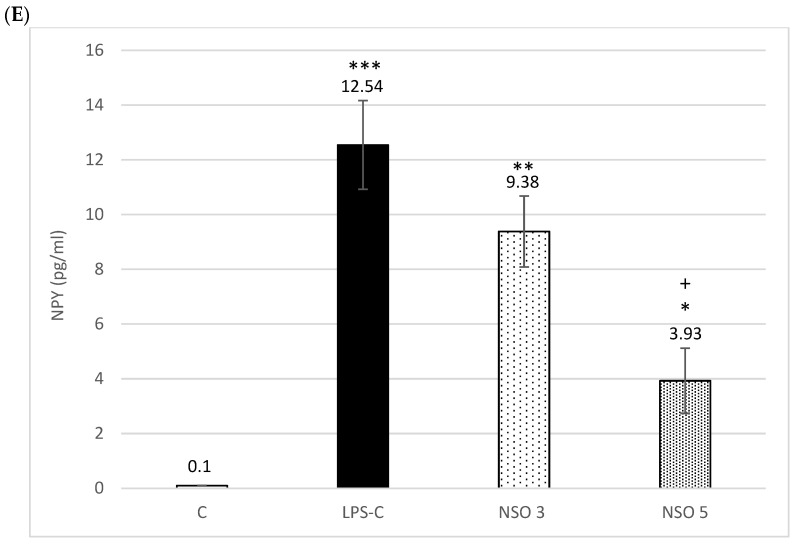
Effects of NSO on serum levels of TNF-α, IL-1β, IL-10, BDNF, and NPY in LPS-challenged rats. (**A**) TNF-α; (**B**) IL-1β; (**C**) IL-10; (**D**) BDNF; (**E**) NPY. C—control group; LPS-C—LPS-control group; NSO-3—group treated with NSO 3 mL/kg BW; NSO-5—group treated with NSO 5 mL/kg BW. Data are expressed as mean ± SEM (n = 8). * *p* < 0.05 compared to C; ** *p* < 0.01 compared to C; *** *p* < 0.001 compared to C; + *p* < 0.05 compared to LPS-C; ++ *p* < 0.01 compared to LPS-C.

The rats from the LPS-treated control group (LPS-C) were detected as having significantly increased levels of TNF-α in comparison to the untreated control (C-group) (*p* < 0.001). In both groups treated with NSO was observed markedly decreased concentration of TNF-α in comparison to the LPS-C group (*p* < 0.05; *p* < 0.01, resp). When compared to the C-group, higher levels of TNF-α were measured (*p* < 0.01) (Figure 2A).

#### 2.2.2. IL-1β

In animals from the LPS-C group, a significantly elevated serum concentration of IL-1β was observed compared to the C-group (*p* < 0.01). In contrast, a significantly decreased IL-1β level was detected in both experimental groups receiving NSO at doses of 3 mL/kg and 5 mL/kg (NSO-3 and NSO-5), compared to the LPS-C group (*p* < 0.05) (Figure 2B).

#### 2.2.3. IL-10

Serum IL-10 levels were significantly elevated in rats from the LPS-C group compared to the C-group (*p* < 0.05). In animals treated with NSO at both doses, a further significant increase in serum IL-10 levels was observed compared to both the C-group (*p* < 0.01) and the LPS-C group (*p* < 0.05) (Figure 2C).

#### 2.2.4. BDNF

A non-significant decrease in BDNF levels was observed in rats from the LPS-C group compared to the C-group. An increase in serum BDNF concentration was measured in both experimental groups; however, statistical significance was reached only in animals treated with the higher NSO dose compared to both control groups (*p* < 0.05 and *p* < 0.01, respectively) (Figure 2D).

#### 2.2.5. NPY

Serum NPY levels were significantly elevated in the LPS-C group compared to the C-group (*p* < 0.001). In animals treated with NSO at a dose of 3 mL/kg, a further increase in NPY serum concentration was observed compared to the C-group (*p* < 0.01). However, in rats receiving the higher NSO dose (5 mL/kg), NPY levels remained elevated compared to the C-group (*p* < 0.05) but were significantly reduced relative to the LPS-C group (*p* < 0.05) (Figure 2E).

### 2.3. Effects of NSO on Learning and Memory in LPS-Challenged Rats

#### 2.3.1. NORT

**Figure 3:**
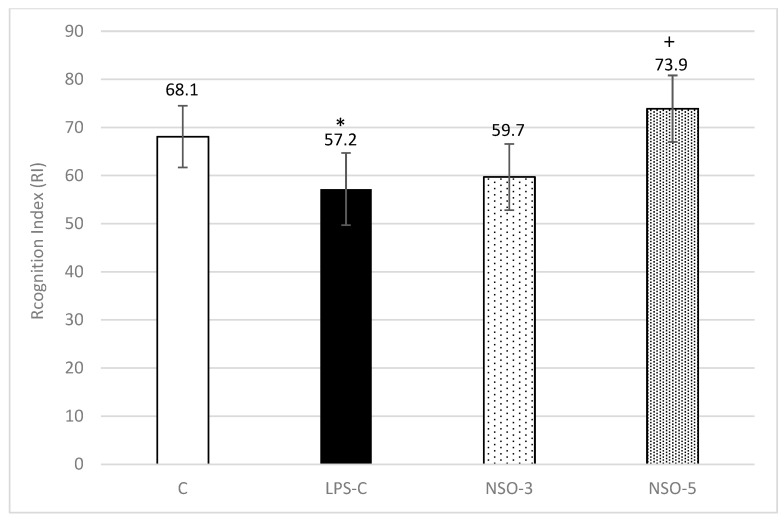
Effect of NSO on RI in LPS-challenged rats. C—control group; LPS-C—LPS-control group; NSO-3—group treated with NSO 3 mL/kg BW; NSO-5—group treated with NSO 5 mL/kg BW. Data are expressed as mean ± SEM (n = 8). * *p* < 0.05 compared to C; + *p* < 0.05 compared to LPS-C.

The recognition index (RI) was significantly decreased in LPS-C rats compared to the C-group (*p* < 0.05). In both experimental groups treated with NSO, an increase in RI was observed compared to the LPS-C group. However, statistical significance was achieved only in the group receiving 5 mL/kg (*p* < 0.05) (Figure 3).

#### 2.3.2. Y-Maze

**Figure 4:**
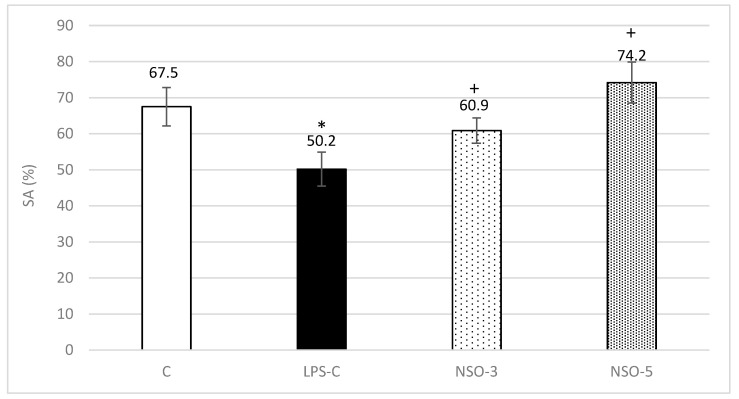
Effect of NSO on SA% in LPS-challenged rats. C—control group; LPS-C—LPS-control group; NSO-3—group treated with NSO 3 mL/kg BW; NSO-5—group treated with NSO 5 mL/kg BW. Data are expressed as mean ± SEM (n = 8). * *p* < 0.05 compared to C; + *p* < 0.05 compared to LPS-C.

In rats from the LPS-C group, a significant decrease in spontaneous alternation percentage (SA%) was observed compared to the C-group (*p* < 0.05). In all animals treated with NSO, a marked increase in SA% was recorded compared to the LPS-C group (*p* < 0.05). Furthermore, rats treated with NSO at a dose of 5 mL/kg BW exhibited higher SA% compared to the C-group (Figure 4).

## 3. Discussion

Our findings showed that treatment with NSO improves spatial working and episodic memory in the LPS-induced model of neurodegeneration and neuroinflammation. These results are consistent with previous studies on the memory-improving effects of NS. Moreover, we found that NS administration ameliorates systemic inflammatory response, which is the trigger of neuroinflammation, by restoring the balance between PICs and IL-10. NSO treatment led to decreased serum NPY in response to the LPS challenge, likely by inhibiting its production in stimulated immune cells. This could be an important mechanism involved in the anti-inflammatory action of NS, which has not been previously investigated.

Systemic LPS administration significantly impaired both episodic and spatial working memory in experimental rats. These results are in line with those reported in our previous studies [42,43], reinforcing the role of neuroinflammation in cognitive deficits. LPS-induced memory impairment likely reflects the impact of neuroinflammatory processes on brain regions critical for memory, such as the hippocampus and prefrontal cortex [44]. LPS can impair various forms of synaptic plasticity, including LTP, in the hippocampus—a key region involved in learning and memory [5]. In the current study, NORT and Y-maze were employed as robust and reliable assessments of hippocampal-dependent learning and memory. These tests are widely recognized for their sensitivity in detecting cognitive impairments related to hippocampal function [45]. Our behavioral studies revealed that NSO effectively prevented LPS-induced cognitive impairments. The animals treated with NSO significantly increased SA% and RI. This suggests that NSO improves spatial working and episodic memory and may mitigate the detrimental effects of LPS, potentially through its anti-inflammatory and neuroprotective properties. The memory-enhancing effects of NS are also largely attributed to its antioxidant properties, which protect neurons from damage caused by oxidative stress, a key factor implicated in cognitive decline and neurodegenerative diseases. One of the main active constituents of NS is TMQ, which has powerful free radical-scavenging capabilities [46]. By neutralizing free radicals, TMQ helps reduce oxidative stress, which in turn prevents neuronal damage and supports brain health overall. The results from the current research showed a relatively high TMQ concentration in the studied oil (21.37 ± 0.38 mg) which, when related to the dosage of the oil per kg bw (3 and 5 mL), corresponds to 64.11 ± 1.14 mg/kg bw and 106.85 ± 1.14 mg/kg bw TMQ. In most studies investigating the biological effects of TMQ, the oral dose varies between 5 and 100 mg/kg body weight, with no signs of toxicity observed, and the maximum tolerated dose being 250 mg/kg body weight. [39,47]. Other compounds found in the oil may also contribute to its memory-improving and neuroprotective effects. Carvacrol improves spatial memory in the Morris water maze and inhibits oxidative stress in the hippocampus of rats with chronic cerebral hypoperfusion [48]. Moreover, in rats with LPS-induced inflammation, carvacrol improves spatial memory in the Morris water maze and decreases lipid peroxidation in the cortex and TNF-α in the hippocampus [49]. Although carvacrol was detected in trace amounts in our NSO, it is important to note that even at low concentrations, it may still contribute to the observed effects, particularly through its potential synergistic interaction with TMQ.

Bacterial lipopolysaccharide is part of the outer membrane of Gram-negative bacteria. Its intraperitoneal administration causes systemic inflammation followed by microglia activation and neuroinflammation [50]. Activated microglia are important factors in the pathogenesis of neurodegenerative disorders, such as AD, PD, amyotrophic lateral sclerosis, etc. [51]. Thus, LPS-induced inflammation is widely used to model neuroinflammation and neurodegeneration in experimental animals. LPS binds to Toll-like receptor 4 (TLR4) in microglial cells and astrocytes and stimulates the production of inflammatory molecules e.g., TNF-α and IL-1β [41]. Single intraperitoneal LPS injection causes a sharp increase in TNF-α and IL-1β serum levels up to 6 h of administration. TNF-α shows peak concentrations in the 2nd hour whereas the highest levels of IL-1β are measured in the 6th hour following LPS-challenged in rats [52]. The results of the current research are consistent with these findings as LPS administration significantly increased serum levels of these PICs in LPS-control. The experimental groups treated with NSO exhibited a significant reduction in serum TNF-α levels, consistent with findings from prior preclinical and clinical studies [53]. Madkour et al. demonstrated that NS mitigated emamectin benzoate-induced neurotoxicity, including the reduction in elevated TNF-α levels in rats [54]. Additional studies have shown that NS lowers inflammatory markers, such as TNF-α and prostaglandin E_2_ (PGE_2_), which are associated with periodontal inflammation [55]. Several clinical studies have confirmed the TNF-α inhibitory effects of NS. Mostafa et al. demonstrated that NSO improved the lipid profile and significantly reduced TNF-α levels in obese prediabetic patients [56]. Another study reported that NS supplementation led to a reduction in TNF-α levels in patients with non-alcoholic fatty liver disease [57].

As previously noted, activated microglia release not only TNF-α but also other pro-inflammatory molecules, including IL-1β. It can initiate a cascade of inflammatory signaling by promoting the synthesis and expression of several secondary cytokines [1]. IL-1β is typically present at low levels in both the CNS and peripheral bloodstream under non-inflammatory conditions. However, elevated levels of IL-1β in the CNS, along with subsequent neuroinflammatory processes, have been reported in various neurological disorders, including multiple sclerosis (MS), AD, and PD [58]. IL-1β has been implicated in directly modulating the nervous system by influencing the levels and activity of a variety of neurotransmitters such as serotonin, gamma-aminobutyric acid (GABA), ACh, and norepinephrine (NE) in different brain regions [5]. LPS and IL-1β have been shown to impair both the induction and maintenance of LTP in the CA1 region and dentate gyrus of the rat hippocampus [59]. This effect may contribute to cognitive deficits observed in models of neuroinflammation. Moreover, the influx of calcium (Ca^2+^) through NMDA (N-methyl-D-aspartate) receptors plays a crucial role in the induction of LTP in the CA1 and CA3 regions of the hippocampus. IL-1β can reduce the activity of L-type voltage-gated calcium channels (VGCCs) and possibly NMDA receptor-mediated calcium entry, both essential for LTP induction [60]. Clinical data demonstrated that higher circulating levels of IL-1β and IL-6 were linked to pronounced deterioration in cognitive abilities [61]. Such evidence strongly suggests that systemic inflammation plays a significant role in modulating cerebral function, both in health and disease. Cerebral endothelial cells (CECs), which form the blood–brain barrier (BBB), are key players in sensing and responding to systemic inflammation due to their expression of cytokine receptors and adhesion molecules on their luminal (blood-facing) surface. TNF-α and IL-1 receptors on CECs, particularly those in brain venules, play a crucial role in mediating systemic inflammatory signals and their effects on the brain. When activated, these receptors trigger a cascade of events that contribute to neuroinflammation, BBB disruption, and cognitive impairment [3]. In our study, NSO significantly decreased the serum concentration of IL-1β, which aligns with other experimental results. Hosseini et al. demonstrated that NSO lowered the plasma levels of TNF-α, PGE2, IL-1β, and IL-6 in LPS-challenged mice [62]. We could assume that NSO by mitigating systemic inflammation reduces the risk of neuroinflammation and its consequences, such as memory impairment. Unexpectedly, our findings demonstrated that the effect of NSO on IL-1β levels was more pronounced in animals treated with the lower dose of NSO, although the difference did not reach statistical significance. This could be related to the existing relationship between IL-1β and TNF-α. Previous studies have shown that IL-1β stimulates the expression of TNF-α receptors and enhances its pro-inflammatory effect [63]. We can speculate that the less pronounced effect of the higher NSO dose on IL-1β may represent a compensatory mechanism in response to the more significant effect of this dose on TNF-α serum levels. Further studies are required to clarify this issue.

The inhibitory effect of NSO on the production of PICs can be attributed to its major active constituent TMQ. It inhibits TNF-α and IL-1β in various experimental models [64]. Arjumand S et al. showed that TMQ possesses the potential to improve rheumatoid arthritis symptoms by downregulating mRNA expression for TLR2, TLR4, TNF-α, IL-1, and NFκB [65]. Carvacrol also has the potential to decrease the production of TNF-α and IL-1β and inhibit protein expression of NFκB [66].

IL-10 regulates inflammation by suppressing the production of PICs. A recent study demonstrated that microglia do not produce IL-10 but express receptors for this cytokine. IL-10 plays an important role in LTP maintenance in the hippocampus and in supporting microglial homeostasis following peripheral LPS administration. The protective role of IL-10 is largely attributed to its ability to suppress the production of TNF-α by activated microglia [67]. Administration of IL-10 ameliorates stress-induced cognitive deficits and improves recognition and spatial memory in experimental animals [68]. NS inhibits the expression of pro-inflammatory mediators and stimulates that of IL-10 in glioma and fibroblast cells [69]. Animal studies demonstrate that NS stimulates IL-10 synthesis in different experimental models [70,71]. In vitro studies with LPS-activated microglial cells found that NS seed oil suppresses the inflammatory response of microglia probably by shifting from M_1_ to M_2_ phenotype, i.e., from production of pro-inflammatory to anti-inflammatory cytokines [62]. The results of the present study provide evidence that NSO likely induces macrophage polarization from M_1_ to M_2_ in the periphery, as low TNF-α and IL-1β and high IL-10 serum levels were observed. This indicates that NSO may maintain an anti-inflammatory state in response to the LPS challenge by restoring the balance between Th_1_ and Th_2_ cytokines. Clinical studies in patients with RA also revealed that NSO increases serum IL-10 levels [72].

LPS binds to TLR4 and causes activation of the NF-kB signaling pathway in the brain resulting in increased synthesis of pro-inflammatory molecules, including TNF-α and IL-1β. This causes microglial activation leading to neuroinflammation and memory impairment [73]. The observed effect of NSO on TNF-α and IL-1β could be due to the inhibitory effect of TMQ and carvacrol on NF-kB activation [65,66].

One of the mechanisms through which inflammation affects the brain is by modulating BDNF expression. It is one of the most important neurotrophins, playing a crucial role in the normal development and functioning of the brain. BDNF is essential for neurogenesis, synaptogenesis, and LTP in the hippocampus and other brain structures. Low BDNF expressions and levels are observed in major depressive disorders, anxiety disorders, and neurodegenerative diseases, such as AD, PD, etc. [13]. Intraperitoneal administration of LPS or exogenous PICs, such as IL-1β, reduces BDNF expression in specific brain regions, including the hippocampus [74]. In the present study, we found that LPS, when administered systemically, decreases serum BDNF. The interpretation of serum BDNF levels is challenging because data regarding its ability to cross the BBB are controversial. Some studies found that this neurotrophin crosses the BBB, while others report little or no permeability. On the other hand, BDNF is produced and released by peripheral tissues and cells, with the platelets being the major source [75]. However, preclinical studies have shown that in rats, BDNF concentrations in the hippocampus correlate with those in the blood, suggesting their potential use as markers for the levels of this neurotrophic factor in this brain structure [76]. Meta-analyses of clinical trials in patients with bipolar disorders showed that peripheral BDNF levels correlate with disease activity [77]. The results of the current study demonstrated that NSO increases serum BDNF levels in LPS-challenged rats. Based on the aforementioned, we can speculate that elevated peripheral BDNF concentrations are accompanied by a parallel increase in hippocampal levels. It remains unclear whether NSO enhances the release of this neurotrophin from peripheral tissues with subsequent transfer through LPS-impaired BBB or whether NSO stimulates BDNF expression in neurons followed by release in peripheral blood. Further studies regarding the effect of NSO on BDNF expression in the brain and its release from platelets are required to clarify this issue.

To our knowledge, this is the first preclinical study that demonstrates the stimulating effect of NSO on peripheral BDNF synthesis and release in the settings of LPS-induced inflammation. In a recent clinical study on patients with depressive disorder, Zadeh AR et al. found that NS extract improves depression scores and increases serum BDNF [78]. The stimulating effect of NSO on BDNF synthesis may at least partially be explained by the presence of TMQ in the oil at high concentrations. A recent study showed that TMQ stimulates BDNF mRNA and protein expression in rat hippocampus [35].

NPY is one of the most prevalent neuropeptides in both the central and peripheral nervous systems. Its expression is highly conserved across different species [79], making experimental data obtained from rodents with a high degree of reliability applicable to humans. NPY has neuroprotective properties, and alterations in its expression are found in neurodegenerative disorders [80]. Studies found that there are low serum and cerebrospinal NPY levels in patients with AD [81]. The neuroprotective effect of this neuropeptide is attributed to various mechanisms, including inhibition of microglia-induced neuroinflammation, reduction in oxidative and mitochondrial damage, stimulation of autophagy, upregulation of neurotrophic factor expression, and suppression of glutamate-induced cytotoxicity [80,81]. NPY is expressed not only in the nervous system but also in peripheral tissues. This neuropeptide is produced by immune cells upon stimulation and plays a role in regulating the immune response. Receptors for NPY are found in almost all types of immune cells. Among these, the Y1 receptor exhibits a dual effect with evidence suggesting that it can both stimulate and inhibit the immune response [82]. Experimental studies with mice lacking the Y1 receptor demonstrate that NPY inhibits T cells, while on the other hand, it is essential for the normal functioning of antigen-presenting cells. Therefore, NPY overproduction could lead to excessive macrophage activation and the promotion of an inflammatory response [83]. In an experimental model of bronchial hyperreactivity and inflammation, NPY is important for the accumulation of CD11c^+^ antigen-presenting cells in the airways and the activation of the Th2 immune response [84]. Thus, while NPY plays a protective role in the CNS, elevated serum levels may have an adverse effect due to their pro-inflammatory action. There is evidence linking high NPY concentrations with an increased risk of cardiovascular and renal damage [85]. In the current study, LPS led to a sharp increase in serum NPY, likely by inducing its production from immune cells. As mentioned above, this neuropeptide is not constitutively expressed in these cells, but its production is stimulated upon activation. Considering that NPY stimulates macrophages, we can speculate that this neuropeptide contributes to and amplifies LPS-induced inflammation. In NSO-treated animals, we observed a significant decrease in serum NPY levels. To the best of our knowledge, there are no reports in the literature regarding the effect of NS or TQN on this neuropeptide. Their impact on NPY synthesis and release from immune cells is likely involved in the observed anti-inflammatory effect. Future studies are needed to evaluate the effect of NSO on brain NPY levels and its relationship with the neuroprotective and memory-enhancing effects of the oil.

The aforementioned results are summarized in Table 2.

Despite the fact that TMQ exerts multiple pharmacological effects and is the primary contributor to the neuroprotective and anti-inflammatory properties of NSO, its clinical application remains unfeasible. TMQ is a hydrophobic compound, a characteristic that significantly limits the development of suitable drug formulations with adequate bioavailability following oral administration. It undergoes slow gastrointestinal absorption, followed by hepatic metabolism into thymohydroquinone and rapid systemic elimination. These pharmacokinetic properties, combined with formulation challenges, pose significant obstacles to its clinical use [20].

To the best of our knowledge, no studies have directly compared the absorption of TMQ from NSO with its absorption when administered as a pure substance. However, we may speculate that the absorption of TMQ from NSO may be enhanced due to its complex phytochemical composition. NSO contains essential fatty acids, terpenes, and other bioactive compounds, which may improve the solubility, stability, and overall bioavailability of TMQ. Previous studies have shown that when TMQ is formulated in lipid-based nanoparticles, its bioavailability is significantly increased, suggesting that lipid formulations can enhance the absorption of this compound [86]. Therefore, the same principle may apply to TMQ in NSO, although further pharmacokinetic studies are necessary to validate this hypothesis.

In this context, our study focused on investigating NSO rather than pure TMQ. NSO is commercially available and may serve as a more practical and accessible therapeutic option, potentially offering adjuvant benefits in the prevention and treatment of diseases associated with low-grade systemic and neuroinflammation.

## 4. Materials and Methods

### 4.1. Drugs and Reagents

Nigella sativa oil (cold pressed black cumin seed oil, Bioherba, Plovdiv, Bulgaria, SKU BH5431); lipopolysaccharide from Escherichia coli serotype O55:B5 (Merck, Darmstadt, Germany, SKU L2880-10MG); rat IL-1β ELISA kit (Diaclone, Besançon, France, Cat. № 670.040.096) rat IL-10 ELISA kit (Diaclone, Besançon, France, Cat. № 670.070.096); rat TNF-α ELISA kit (Diaclone, Besançon, France, Cat. № 805.000.096), rat BDNF ELISA kit (Abcam, Cambridge, UK, ab213899) and rat NPY ELISA kit (Cusabio, Wuhan, China, Cat. № CSB-E13431r); thymoquinone for analytical standard (Merck, Darmstadt, Germany, cat. № 03416-100MG); thymol (Merck, Darmstadt, Germany, cat. № 72477-500MG) and carvacrol (Merck, Darmstadt, Germany, cat. № 42632-50MG).

### 4.2. HPLC Analysis of NSO

The preparation of the sample for analysis consisted of obtaining one hundred and one thousand times diluted solutions of NSO in hexane, which were filtered through a microfilter (0.45 μm). The HPLC system was composed of a ProStar 230 solvent delivery module with photodiode array detector model 335 (Varian, Belrose, Australia), and a Hitachi C18 AQ (250 mm × 4.6 mm, 5 μm) column (Hitachi, Tokyo, Japan). For the separation of thymoquinone, thymol, and carvacrol, the mobile phase H_2_O (A) with pH 3.0 (achieved with H_3_PO_4_) and 40 acetonitrile/60 methanol (B) was used in the gradient mode shown in Table 3:

The flow rate was 1.0 mL/min and the injection volume was 20 µL. Detection of substances was carried out at 254 nm for thymoquinone and at 275 nm for thymol and carvacrol at which their maximum absorption was. The chromatogram was recorded at 254 nm until 17.5 min and at 275 nm until the end. The target compounds were quantified using a calibration curve. The identification of substances was carried out both by retention times and by comparing their absorption spectra with those of standards. Correlation coefficients (r) were also calculated between standard spectra and the spectra of the samples based on the formula:

(1) $$r=\frac{\sum (a_{i}-\left.\bar{a}\right)\left(b_{i}-\bar{b})\right.}{\sqrt{\sum (a_{i}-{\left.\bar{a}\right)}^{2}\sum {\left(b_{i}-\bar{b})\right.}^{2}}}\;$$

where *a_i_* and *b_i_* are the absorbance values at the *i*th wavelength.

Star Chromatography Workstation Version 6.30 (build 5) software was used for data processing.

### 4.3. Animals

In the current study, we used adult male rats of Wistar strain (200 ± 20 g—body weight (bw)). They were housed under controlled laboratory conditions (12 h light-dark cycles, room temperature 22 ± 2 °C, air humidity 55 ± 5%). Water and food were given ad libitum. The experiments were run during the daytime and the animals were accustomed to the laboratory environment prior to the tests.

### 4.4. Experimental Design

Animals were randomly divided into 4 groups (n = 8):

Group 1: control group (C): olive oil 1 mL/kg BW;

Group 2: LPS-control group (LPS-C): olive oil 0.1 mL/100 g BW + LPS 2 mg/kg BW;

Group 3: NSO 3 mL/kg BW + LPS 2 mg/kg BW (NSO-3);

Group 4: NSO 5 mL/kg BW + LPS 2 mg/kg BW (NSO-5).

All animals were treated orally once daily for 3 weeks prior to the behavioral tests and throughout the entire experimental trial. Control animals were treated with olive oil instead of saline due to the oily nature of the investigated substance, making olive oil an appropriate comparator. To minimize potential confounding effects from bioactive compounds, we selected commercially available refined olive oil, as its polyphenolic content is negligibly low [87]. The doses of NSO were determined based on available literature data on the LD_50_ (28.8 mL/kg) [33]. In the current experiment, we used 1/10 and 1/6 of LD_50_. LPS was injected intraperitoneally in a dose of 2 mg/kg BW on day 15 to groups 2, 3, and 4. Blood samples for immunological assay were collected 4 h later as inflammatory molecule levels rose rapidly following LPS administration but returned to baseline within hours. However, neuroinflammation developed at a later stage. Therefore, behavioral tests were conducted 7 days after the LPS challenge [52].

### 4.5. Behavioral Tests

#### 4.5.1. NORT and Y-Maze Were Used to Assess Learning and Memory Processes

NORT was employed to assess exploratory behavior and recognition memory, which are components of explicit memory in rodents. The experimental setup included an open acrylic box measuring 60 cm in length, 60 cm in width, and 40 cm in height. The trial was conducted over two consecutive days and comprised three phases: habituation, investigation, and testing.

During the habituation phase, the rats were allowed to acclimate to the test chamber for 5 min without any objects present. Following this, the rats were placed back in the same chamber with two identical objects for an additional 5 min to facilitate investigation.

Twenty-four hours later, a memory test was performed. In this phase, one of the identical objects was replaced with a novel object, and the rats were given 5 min to explore the chamber. The time spent exploring the novel (N) and familiar (F) objects was recorded. RI was calculated using the following formula:

RI = N/(N + F) × 100

where N is the time for exploration of the novel item, and F is the time for exploration of the familiar one.

#### 4.5.2. Y-Maze Is Generally Used to Evaluate Spatial Working Memory in Rodents

That test is based on the natural exploratory instinct of the rodents. The experimental set presents three plexiglass arms (length 50 cm, width 10 cm, and height 30 cm) interconnected at 120°. The arms were randomly labeled A, B, and C. On two consecutive days, spontaneous alternation tests were performed: a training session on day 1 and a memory retention test on day 2. The rats were placed in the center of the setup and allowed to explore the arms for 5 min. Consecutive entry into the three arms of the maze was considered as alternation, e.g., ABC, BCA, CAB, CBA, etc. SA% is measured to evaluate the rodents’ ability to remember previous choices and explore new areas. The formula for calculating spontaneous alternation is as follows:

SA% = NA/(TNE-2) × 100

NA—number of alterations (i.e., the number of times the rat entered all three arms in succession); TNE—total number of entries (the total entries made by the rat into any arm).

### 4.6. Sample Collection

For the collection of blood samples, we used pyrogen and endotoxin-free tubes. Blood samples were centrifuged for 10 min, serum separated, and stored at −70 °C. Serum levels of TNF-α, IL-1β, IL-10, BDNF, and NPY were measured with solid-phase ELISA. Absorbance was read at 450 nm and recalculated as concentrations (pg/mL).

### 4.7. Statistical Analysis

We used IBM SPSS Statistics 19.0 for the analysis. All data were presented as mean ± SEM. The differences between the groups were analyzed by one-way ANOVA, followed by Tukey pos hoc test for comparison between the experimental groups. The value of *p* < 0.05 was considered statistically significant.

## 5. Conclusions

Emerging studies have increasingly highlighted the immunomodulatory and anti-inflammatory properties of NSO and its bioactive components, particularly TMQ. Our findings showed that NSO modulates inflammation by lowering serum levels of key PICs, including TNF-α, and IL-1β, and increasing IL-10 levels in an experimental model of systemic and neuroinflammation. Since these effects are typically associated with TMQ, which is the main biologically active compound in the oil, we developed an HPLC method for its validation. TMQ quantification in NSO-based nutritional supplements is crucial, considering its instability under aversive conditions. Additionally, for the first time to our knowledge, we found that the immunomodulatory effect of the oil may be attributed to its action on the levels of NPY in peripheral tissues, which provides a new direction for research in this field. Bearing those findings in mind, it is likely that the improvement of memory in the context of LPS-induced impairments is thought to be, at least partially, due to NSO acting as a potent anti-inflammatory and immunomodulatory agent. Therefore, NSO stands out as a promising natural supplement for improving cognitive function.

## Figures and Tables

**Table 1:** Parameters of calibration curves, RSD, LOD, and LOQ for HPLC method validation.

Analyte	λ (nm)	RT (min)	Regression Equations	r^2^	RSD (%)	LOD (µg/mL)	LOQ (µg/mL)	r
Thymoquinone	254	16.96	y = 3.5463e + 005x	0.9974	5.92	0.640	1.935	0.9991
Thymol	275	18.31	y = 1.5218e + 005x	0.9991	3.03	0.215	0.645	-
Carvacrol	275	18.56	y = 1.5941e + 005x	0.9996	1.77	0.380	1.148	0.9989

**Table 2:** Effects of NSO on serum levels of TNF-α, IL-1β, IL-10, BDNF, and NPY in LPS-challenged rats.

	Doses	NSO 3 mL/kg	NSO 5 mL/kg
Biological Marker			
TNF-α		398.7 ± 36.7 **^+^	247.4 ± 17.9 **^++^
IL-1β		40.02 ± 8.22 ^+^	64.52 ± 22.13 ^+^
IL-10		458.7 ± 45.3 **^+^	468.4 ± 42.6 **^+^
NPY		9.38 ± 1.3 **	3.93 ± 1.19 *^+^
BDNF		1258.31 ± 154.27	1780.75 ± 210.17 *^++^

Data are expressed as mean ± SEM (n = 8). * *p* < 0.05 compared to C; ** *p* < 0.01 compared to C; ^+^ *p* < 0.05 compared to LPS-C; ^++^ *p* < 0.01 compared to LPS-C.

**Table 3:** Mobile phase gradient.

	Time, min	Initial	7	14	20	23	25
Phase, %							
A		50	50	20	10	50	50
B		50	50	80	90	50	50

## Data Availability

The data presented in this study are available on request from the corresponding author.

## Abbreviations

The following abbreviations are used in this manuscript:

AD	Alzheimer’s disease
BBB	Blood–brain barrier
BDNF	Brain-derived neurotrophic factor
Ca^2+^	Calcium
CECs	Cerebral endothelial cells
CNS	Central nervous system
GABA	Gamma-aminobutyric acid
LPS	Lipopolysaccharide
LTP	Long-term potentiation
MS	Multiple sclerosis
NE	Norepinephrine
NMDA	N-methyl-D-aspartate
NORT	Novel object recognition test
NPY	Neuropeptide Y
NS	Nigella sativa
NSO	Nigella sativa oil
PD	Parkinson’s disease
PGE_2_	Prostaglandin E_2_
PIC_s_	Pro-inflammatory cytokines
RI	Recognition index
SA%	Spontaneous alternation %
TLR4	Toll-like receptor4
TMQ	Thymoquinone
VGCC_S_	Voltage-gated calcium channels
